# Drifted Influenza A and B Viruses Collected by a Water-Based Condensation Growth Air Sampler in a Student Health Care Center during an Influenza Outbreak

**DOI:** 10.1128/genomeA.00178-17

**Published:** 2017-04-13

**Authors:** Tania S. Bonny, Maohua Pan, Julia C. Loeb, Xiao Jiang, Arantzazu Eiguren-Fernandez, Susanne Hering, Z. Hugh Fan, Chang-Yu Wu, John A. Lednicky

**Affiliations:** a Department of Environmental and Global Health, College of Public Health and Health Professions, University of Florida, Gainesville, Florida, USA; b Emerging Pathogens Institute, University of Florida, Gainesville, Florida, USA; c Department of Environmental Engineering Sciences, Engineering School of Sustainable Infrastructure and Environment, University of Florida, Gainesville, Florida, USA; d J. Crayton Pruitt Family Department of Biomedical Engineering, University of Florida, Gainesville, Florida, USA; e Aerosol Dynamics, Inc., Berkeley, California, USA; f Department of Mechanical and Aerospace Engineering, University of Florida, Gainesville, Florida, USA

## Abstract

A viable virus aerosol sampler (VIVAS) effectively collected viable influenza A and B viruses from air inside a student health care center during an influenza outbreak. The viruses had “drifted” genes, showcasing the usefulness of the VIVAS for air sampling and noninvasive surveillance of viruses in circulation.

## GENOME ANNOUNCEMENT

The dynamics and significance of aerosol transmission of human influenza viruses remain controversial (1, 2). A major obstacle has been that virus aerosols are not easily collected by commonly used air samplers, and the viruses collected by such devices are often inactivated by the collection method (3). Infection risk analyses lack accuracy when they are based on virus detection alone, as breathing air always contains viruses that have been inactivated through exposure to ultraviolet light, drying, or other means, and thus pose no inhalation hazards. We have been evaluating and refining the viable virus aerosol sampler (VIVAS), a novel air sampler that operates on a water vapor condensation process to enlarge aerosolized virus particles to facilitate their capture (3, 4), and we tested it thrice a day on three different days during an influenza outbreak. Air samplings were performed in March and April 2016 at the Student Health Care Center (SHC) of the University of Florida; the study was exempt from institutional review board (IRB) approval and was approved by the SHC’s director.

The quantity of viruses collected in air samplers is typically low and below the detection limit of PCR-based methods. To improve the chances of virus detection, virus isolation was attempted by inoculation of aliquots of the collection medium onto a variety of cell lines. Remarkably, mixed virus-induced cytopathic effects (CPE) were observed in various cell lines, indicating the isolation of different types of viruses on two of the test days (only human metapneumovirus was isolated on the third attempt).

Typical influenza virus (IFV) CPE, including the formation of focal enlarged granular cells, followed by nonspecific cell deterioration and detachment of the swollen cells from the growth surface, were observed in Madin-Darby canine kidney (MDCK) cell monolayer cultures. To quickly screen for the presence of influenza A and B virions, the spent medium of MDCK cell cultures that exhibited typical IFV-induced CPE was tested using a commercial solid-phase enzyme-linked immunosorbent assay (ELISA) (QuickVue influenza A and B kit; Quidel Corp., San Diego, CA, USA) (1, 5). Viral RNA was purified from the virus particles in the medium, and real-time PCR (RT-PCR) analyses were performed to establish virus type and subtype. Both pdmH1N1 and H3N2 IFV A viruses were recovered, as was Victoria lineage IFV B virus. Sanger sequencing of influenza A virus genomic segments 4 (hemagglutinin [HA] gene), 6 (neuraminidase [NA] gene), and 7 (matrix [M2 and M1] genes) and influenza B virus segments 4 (HA gene), 6 (NB glycoprotein [NB] and NA genes), and 7 (matrix protein 1 [M1] and BM2 genes) was accomplished according to previously described methods (1, 6, 7).

Sequencing revealed that the pdmH1N1 viruses belonged to hemagglutinin (HA) subclade 6B.1 (8–10), the H3N2 viruses belonged to HA clade subclade 3C.2a (7, 8), and the IFV B virus was a Victoria lineage clade 1A virus (11). Of interest, the viruses we isolated differ from those of the trivalent Northern Hemisphere influenza virus vaccines for 2015 to 2016, which contained a clade 1 pdmH1N1, A/California/7/2009 (H1N1); a clade 3C.3a H3N2, A/Switzerland/9715293/2013 (H3N2); and a clade 3 Yamagata-lineage IFV B (a B/Phuket/3073/2013) virus.

We conclude that the VIVAS could be useful for passive noninvasive virus surveillance applications.

### Accession number(s).

The sequences of this study have been deposited in the GenBank database under the following accession numbers: IFV pdmH1N1 HA gene, KX398060, KX398061, KX398062, and KX398063; IFV pdmH1N1 NA gene, KX398064, KX398065, KX398066, and KX398067; IFV pdmH1N1 M2 and M1 genes, KX398068, KX398069, KX398070, and KX398071; IFV H3N2 HA gene, KX398081, KX398082, and KX398083; IFV H3N2 NA gene, KX398084, KX398085, and KX398086; IFV H3N2 M2 and M1 genes, KX398087, KX398088, and KX398089; IFV B HA gene, KX398072, KX398073, and KX398074; IFV B NB and NA genes, KX398075, KX398076, and KX398077; and IFV B M1 and BM2 genes, KX398078, KX398079, and KX398080.

## References

[1] Lednicky JA, Loeb JC 2013 Detection and isolation of airborne influenza A H3N2 virus using a Sioutas personal cascade impactor sampler. Influenza Res Treat 2013:656825. doi:10.1155/2013/656825. 24224087 PMC3810434

[2] Killingley B, Nguyen-Van-Tam J 2013 Routes of influenza transmission. Influenza Other Respir 7(Suppl 2):42–51. https://doi.org/10.1111/irv.12080 10.1111/irv.12080 PMC5909391 24034483

[3] Lednicky JA, Pan M, Loeb JC, Hsieh H, Eiguren-Fernandez A, Hering SZ, Fan ZH, Wu CY 2016 Highly efficient collection of infectious pandemic influenza H1N1 virus (2009) through laminar-flow water based condensation. Aerosol Sci Technol 50:i–iv. doi:10.1080/02786826.2016.1179254.

[4] Pan M, Eiguren-Fernandez A, Hsieh H, Afshar-Mohajer N, Hering SV, Lednicky JA, Hugh Fan Z, Wu CY 2016 Efficient collection of viable virus aerosol through laminar-flow, water-based condensational particle growth. J Appl Microbiol 120:805–815. doi:10.1111/jam.13051. 26751045 PMC10720391

[5] Lednicky JA, Hamilton SB, Tuttle RS, Sosna WA, Daniels DE, Swayne DE 2010 Ferrets develop fatal influenza after inhaling small particle aerosols of highly pathogenic avian influenza virus A/Vietnam/1203/2004 (H5N1). Virol J 7:231. doi:10.1186/1743-422X-7-231. 20843329 PMC2949836

[6] Iovine NM, Morris JG Jr, Fredenburg K, Rand KH, Alnuaimat H, Lipori G, Brew J, Lednicky JA 2015 Severity of influenza A(H1N1) illness and emergence of D225G variant, 2013–14 influenza season, Florida, USA. Emerg Infect Dis 21:664–667. doi:10.3201/eid2104.141375. 25811540 PMC4378462

[7] Lednicky JA, Iovine NM, Brew J, Loeb JC, Sugimoto JD, Rand KH, Morris JG Jr 2016 Hemagglutinin gene clade 3C.2a influenza A(H3N2) viruses, Alachua County, Florida, USA, 2014–15. Emerg Infect Dis 22:121–123. doi:10.3201/2201.151019. 26692074 PMC4696699

[8] European Centre for Disease Prevention and Control (ECDC) 2016 Surveillance report: influenza virus characterisation. Summary Europe, March 2016. European Centre for Disease Prevention and Control, Stockholm, Sweden http://ecdc.europa.eu/en/publications/Publications/influenza-virus-characterisation-march-2016.pdf

[9] Parida M, Dash PK, Kumar JS, Joshi G, Tandel K, Sharma S, Srivastava A, Agarwal A, Saha A, Saraswat S, Karothia D, Malviya V 2016 Emergence of influenza A (H1N1) pdm09 genogroup 6B and drug resistant virus, India, January to May 2015. Euro Surveill 21:6–11. doi:10.2807/1560-7917.ES.2016.21.5.30124. 26876980

[10] Broberg E, Melidou A, Prosenc K, Bragstad K, Hungnes O; WHO European Region and the European Influenza Surveillance Network members of the reporting countries 2016 Predominance of influenza A(H1N1)pdm09 virus genetic subclade 6B.1 and influenza B/Victoria lineage viruses at the start of the 2015/16 influenza season in Europe. Euro Surveill 21:pii=30184. doi:10.2807/1560-7917.ES.2016.21.13.30184. 27074657

[11] European Centre for Disease Prevention and Control 2013 Surveillance report: influenza virus characterisation. Summary Europe, June 2013 http://ecdc.europa.eu/en/publications/Publications/influenza-virus-characterisation-june-2013.pdf.

